# Increasing cell–device adherence using cultured insect cells for receptor-based biosensors

**DOI:** 10.1098/rsos.172366

**Published:** 2018-03-21

**Authors:** Daigo Terutsuki, Hidefumi Mitsuno, Takeshi Sakurai, Yuki Okamoto, Agnès Tixier-Mita, Hiroshi Toshiyoshi, Yoshio Mita, Ryohei Kanzaki

**Affiliations:** 1 Department of Advanced Interdisciplinary Studies, Graduate School of Engineering, The University of Tokyo, 4-6-1 Komaba, Meguro-ku, Tokyo, Japan; 2 Research Center for Advanced Science and Technology (RCAST), The University of Tokyo, 4-6-1 Komaba, Meguro-ku, Tokyo, Japan; 3 Department of Electrical Engineering and Information Systems, Graduate School of Engineering, The University of Tokyo, 7-3-1 Hongo, Bunkyo-ku, Tokyo, Japan

**Keywords:** cell–electronic interface, cell adhesion, sf21 insect cell, odorant sensor, field-effect transistor, cross-section polisher

## Abstract

Field-effect transistor (FET)-based biosensors have a wide range of applications, and a bio-FET odorant sensor, based on insect (Sf21) cells expressing insect odorant receptors (ORs) with sensitivity and selectivity, has emerged. To fully realize the practical application of bio-FET odorant sensors, knowledge of the cell–device interface for efficient signal transfer, and a reliable and low-cost measurement system using the commercial complementary metal-oxide semiconductor (CMOS) foundry process, will be indispensable. However, the interfaces between Sf21 cells and sensor devices are largely unknown, and electrode materials used in the commercial CMOS foundry process are generally limited to aluminium, which is reportedly toxic to cells. In this study, we investigated Sf21 cell–device interfaces by developing cross-sectional specimens. Calcium imaging of Sf21 cells expressing insect ORs was used to verify the functions of Sf21 cells as odorant sensor elements on the electrode materials. We found that the cell–device interface was approximately 10 nm wide on average, suggesting that the adhesion mechanism of Sf21 cells may differ from that of other cells. These results will help to construct accurate signal detection from expressed insect ORs using FETs.

## 1. Introduction

Field-effect transistor (FET)-based biosensors, in which biological cells or receptors are immobilized onto the electrodes, have attractive characteristics such as non-invasive measurement, long-term recording, and high sensitivity and selectivity for environmental monitoring, drug screening and point-of-care testing [1]. Recently, odorant biosensing techniques, based on mammalian or insect odorant receptors (ORs), have emerged [2]. We proposed a new bio-hybrid electronic odorant sensor, termed the odour-sensitive field-effect transistor (OSFET) [3], based on extended-gate FETs with thin Al_2_O_3_ layers and Sf21 insect cells derived from pupal ovarian tissues of *Spodoptera frugiperda* [4] expressing insect ORs. Sf21 cells with insect ORs act as odorant sensor elements [5,6]; simply attaching them to extended-gate electrodes allowed the sensors to discriminate between two structurally similar odorants by electrical signals.

To fully realize the practical uses of OSFETs, it is essential to efficiently detect signals from Sf21 cells expressing insect ORs and to develop a reliable and low-cost measurement system. The signal detection of OSFETs is affected by the adhesive interfaces, which are called clefts, between Sf21 cells expressing insect ORs and the extended-gate electrode surfaces. These clefts disperse the ionic current generated by the cells and thereby cause degradation of the signal-to-noise ratio [7]. Although various cell–device interfaces, such as those of HEK293 cells and some types of neurons, were previously observed by optical methods or electron microscopy [8,9], this knowledge cannot directly be applied to Sf21 cells because of the differences in cell types and shapes. To our knowledge, the adhesive interfaces between Sf21 cells and planar metal or oxide substrates have not been evaluated.

Applying commercial complementary metal-oxide semiconductor (CMOS) foundry processes provided us with reliable electron devices using proved fabrication procedures [10] and led to repeatable measurement results using OSFETs [3]. Thus, CMOS foundry processes have high yields and help to develop cost-effective bio-FET odorant sensors. However, electrode materials in these processes are generally limited to aluminium [11], for which neuro-toxicity has been reported [11–13], and thus, requires costly biocompatible coatings. Therefore, we investigated the compatibility of Sf21 cells with the materials of CMOS devices, in particular, aluminium and Al_2_O_3_ layers.

In this study, we observed and analysed the adhesive interfaces between Sf21 cells expressing insect ORs and aluminium-based layers, including aluminium and Al_2_O_3_. We developed cross-sectional specimens of Sf21 cells using a cross-section polisher (CP), because this method allowed us to develop high-quality cross sections of soft–hard composite specimens with minimal invasion of the specimen structures. Various types of cells are known to form focal adhesions when connecting to substrates [14]. Focal adhesions are characterized by distances of 10–20 nm between the plasma membranes and substrates [15], and in the case of HEK293 cells, 5–20% of adhesive interfaces are estimated to be focal adhesions [9]. By contrast, our observations of the adhesive interfaces using scanning electron microscopy (SEM) suggested that Sf21 cells expressing insect ORs had much closer contact sites than other cells. Cleft states are also strongly related to the electrical models of signal detections, and their distances should ideally be as short as possible for better electrical coupling [16]. Detailed evaluation of the cell–device interfaces in this study will support construction of the electrical model of OSFETs for high-fidelity data acquisition. To verify the functions of Sf21 cells as odorant sensor elements on electrode materials used in commercial CMOS foundry processes, we conducted calcium imaging of Sf21 cells expressing insect ORs on four types of substrates. Additionally, we compared Sf21 and HEK293T cell growth on aluminium layers to understand the effects of aluminium on these cells.

## 2. Material and methods

### 2.1. Odour-sensitive field-effect transistor device chips

**Figure 1.**
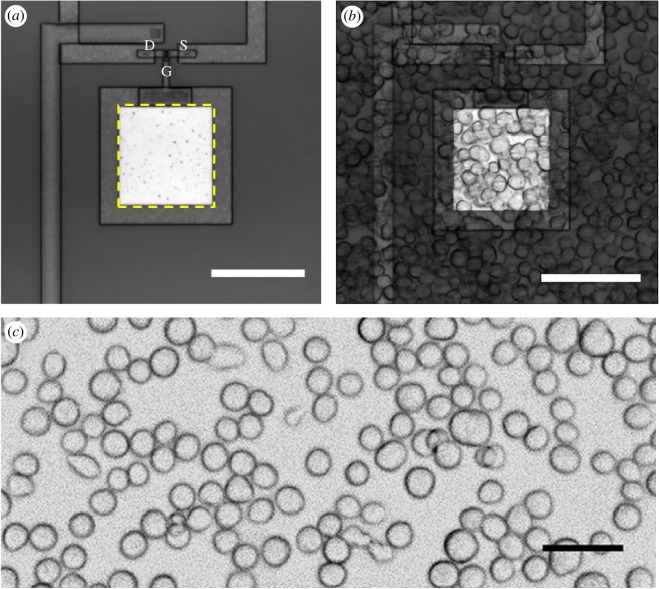
(*a*) Photographs of the developed extended-gate FET chip of the OSFET. The areas of the extended-gate electrodes are 100 × 100 µm^2^. D, drain; S, source; G, gate. The sensing area is inside of the yellow-dashed line. (*b*) OSFET after seeding Sf21 cells expressing insect ORs on its electrode. The cells were incubated at approximately 20°C. (*c*) Bright-field microscopy image of Sf21 cells on plastic cell-culture dish. Scale bars: (*a*) 100 µm, (*b*) 100 µm and (*c*) 50 µm.

We developed extended-gate FETs composed of sensing electrodes and metal-oxide semiconductor field-effect transistor (MOSFET) structures to constitute a part of the OSFET [3]. The gate electrodes extended from the FET gates, and their sensing areas were 100 × 100 µm^2^. Bright-field microscopy images of an extended-gate FET and Sf21 cells attached are shown in figure 1*a*,*b*. The surfaces of the devices, except for the sensing areas, were covered with a SiO_2_ passivation layer. An approximately 40–80 nm Al_2_O_3_ thin film layer was sputtered onto the extended-gate electrodes to stabilize their surface potentials [17].

### 2.2. Cells and odorant

We used Sf21 cells and Sf21 cells expressing insect ORs with OR co-receptors and fluorescent calcium indicator proteins (GCaMP6s) [6]. A bright-field microscopy image of Sf21 cells on a plastic cell-culture dish is shown in figure 1*c*. The cells were cultured in Grace's insect medium (Invitrogen, Carlsbad, CA, USA) at 27°C. A silk moth pheromone receptor BmOR3, which specifically responds to a silk moth pheromone component bombykal ((*E*,*Z*)-10,12-hexadecadienal) [18], was expressed in Sf21 cells. Bombykal was kindly provided by Dr Shigeru Matsuyama (Tsukuba University, Ibaraki, Japan) and was diluted with assay buffer solution (140 mM NaCl, 5.6 mM KCl, 4.5 mM CaCl_2_, 11.26 mM MgCl_2_, 11.32 mM MgSO_4_, 9.4 mM d-glucose, 5 mM HEPES, pH 7.2) containing 0.1% dimethyl sulfoxide (DMSO; Wako Pure Chemical Industries, Ltd, Osaka, Japan) as an odorant. We used Sf21 cells expressing insect ORs for SEM observation of the OSFET. In this study, Sf21 cells expressing BmOR3 are called BmOR3 cells.

HEK293T cells were used for comparing the cell growth on aluminium layers. The cell-culture protocols from the GE Healthcare technical manual (Technical manual, HEK293T Cell Line; GE Healthcare, Chicago, IL, USA) were followed. HEK293T cells and all cell-culture materials were purchased from GE Healthcare. We chose HEK293T cells for comparison because HEK293 cells have been used for biosensors [9], but are not derived from insects. HEK293T cells were cultured in cell-culture flasks with 5 ml Dulbecco's modified Eagle's medium supplemented with 10% defined foetal bovine serum, 6 mM l-glutamine, 100 unit ml^−1^ penicillin and 100 µg ml^−1^ streptomycin in a 5% CO_2_ incubator at 37°C. After reaching confluence, the HEK293T cells were removed with trypsin (0.25% (1×) solution), and 50 µl cell suspensions were seeded on the aluminium substrates in 35 mm plastic cell-culture dishes with 2 ml culture medium in a 5% CO_2_ incubator at 37°C.

### 2.3. Calcium imaging

The activity of BmOR3 cells cultured on four different substrates was evaluated by calcium imaging in the presence of odorant stimulation at different concentrations. The four substrates were: 5 × 5 mm^2^ aluminium-sputtered silicon substrates, Al_2_O_3_-sputtered silicon substrates, bare silicon substrates and 12 mm diameter cover glasses (CS-12R; Warner Instruments, LLC, Hamden, CT, USA); the three types of silicon substrates were placed on 12 mm cover glasses, and plain cover glasses served as control substrate. The substrates with cultured BmOR3 cells were inserted into an open bath chamber (RC-48LP; Warner Instruments, LLC) under a fluorescence microscope. The chamber was installed in a handmade acrylic resin holder with inlet and outlet silicon tubes. Silicon tubes of 1 mm inner diameter were connected to peristaltic pumps that circulated the assay buffer solution including 0.1% DMSO at a flow rate of approximately 1.5 ml min^−1^. Each odorant stimulation was applied for 15 s with the assay buffer solution including 0.1% DMSO. To record the odorant responses of the BmOR3 cells as fluorescence signals, a microscope (BX51WI; Olympus, Tokyo, Japan) with a fluorescence mirror unit for GFP (U-MGFPHQ; Olympus) and a charge-coupled device camera (DU-897E; Andor Technology PLC, Belfast, UK) were used. Matlab (Mathworks, Natick, MA, USA) was used to select cells in the chamber as the region of interest (ROI) based on circular Hough transform [19,20] after obtaining video images of the fluorescence measurements. The fluorescence intensity change was defined as

2.1 $$\frac{\Delta F}{F_{0}}=\frac{F_{t}-F_{0}}{F_{0}},$$

where, *F_t_* is the fluorescence intensity at time *t*, and *F*_0_ is the average fluorescence intensity measured during the 20 s baseline period. The average fluorescence intensity was defined by subtracting the 20 s average of the peak value from the baseline value.

### 2.4. Specimen preparation for scanning electron microscopy observations

BmOR3 cells in 2 ml suspensions were seeded on the OSFET device chips in 35 mm plastic cell-culture dishes (Corning, Inc., Corning, NY, USA) and incubated at approximately 20°C for 30 min. The cell density was adjusted for sparse seeding onto the Al_2_O_3_ layers to enable observation of entire cells and to prevent overlap.

Specimens were prepared stepwise for bird's-eye SEM observations as follows. (1) *Pre-fixation*: the suspensions in cell-culture dishes were removed after 30 min of incubation, and the attached cells were fixed with 0.2 M phosphate-buffered saline (PBS) (Na_2_HPO-NaH_2_PO_4_ mixture: Wako Pure Chemical Industries, Ltd, Osaka, Japan) including 2.5% glutaraldehyde for approximately 3 h. (2) *Washing*: the specimens were washed with 0.1 M PBS. (3) *Post-fixation*: the attached BmOR3 cells were fixed using 2% OsO_4_ (TAAB Laboratories Equipment Ltd, Berkshire, UK) for approximately 1 h. (4) *Dehydration*: the specimens were sequentially immersed in increasing concentrations of ethanol (50%, 70%, 90%, 100%, each for 10 min). (5) *Drying*: critical-point drying, which is a well-established drying technique, was used to minimize shrinkage of the attached BmOR3 cells. (6) *Conductive treatment*: the attached BmOR3 cells were coated with a 10 nm Pt layer by sputtering.

For cross-sectional images, steps (1) to (3) were the same as those for the bird's-eye SEM images, and a 10 nm carbon coating was applied by ion beam sputtering. The remaining steps were as follows. (4) *Dehydration and replacement*: after sequential immersion in an ethanol gradient as described above, the insect medium was replaced with propylene oxide (Nisshin EM Co., Ltd, Tokyo, Japan). (5) *Resin-embedding*: the specimens were embedded in epoxy resin (EPON812). (6) *Cross-sectioning*: this study employed a CP (IB-19520CCP; JEOL, Akishima, Tokyo, Japan) for high-quality specimen preparation.

### 2.5. Image analysis of cross-sectional specimens

We defined the cleft distances between the attached BmOR3 cells and the Al_2_O_3_ layers by the sharp brightness value that decreases in the ImageJ plot profiles (http://imagej.nih.gov/ij/), and discussed these distances based on the full width at half maximum (FWHM), which has previously been used for plasma-membrane and nanoparticle analyses [21,22]. The plasma membranes of the attached BmOR3 cells were enhanced using a band-pass filter, and smooth/sharpen filters were alternately applied three times to reduce noise. We then analysed the cleft distances using the ImageJ plot profile command with 1 pixel profile line width. The line was set to 1 pixel (=1.86 nm). We drew vertical plot profile lines at 10 pixel intervals using ImageJ macro, and measured their FWHM values using Matlab. Details of the measured points are shown in electronic supplementary material, figure S1. We referred the code posted in ImageJ forum (http://forum.imagej.net/) to write the macro program, and modified only the pixel distance from 50 to 10. The plots obtained by ImageJ macro were fitted using the Piecewise Cubic Hermite Interpolating Polynomial (PCHIP) [23] in Matlab to calculate FWHM values.

## 3. Results

### 3.1. Calcium imaging of BmOR3 cells and cell adhesion on aluminium-based materials

**Figure 2.**
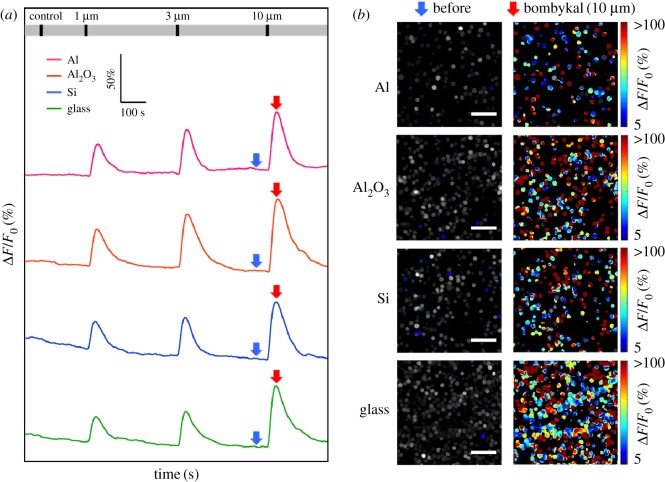
(*a*) Typical dose–response profiles of fluorescence intensity changes of BmOR3 cells binding to aluminium-sputtered silicon substrate, Al_2_O_3_-sputtered silicon substrate, bare silicon substrate and 12 mm diameter cover glasses during the addition of bombykal at concentrations ranging from 1 to 10 µM. These responses indicated average numbers of BmOR3 cells in the fluorescence-microscope field of view. The numbers of attached BmOR3 cells were 165 on the aluminium-sputtered silicon substrate, 363 on the Al_2_O_3_-sputtered silicon substrate, 268 on the bare silicon substrate and 336 on the 12 mm cover glass. The long grey bar indicates the perfusion of the assay buffer solution including 0.1% DMSO by the pumps. The short black bars indicate the times that the odorant stimulations (assay buffer solution including 0.1% DMSO as a control and 1, 3, 10 µM bombykal) flowed inside the chamber for 15 s. (*b*) Pseudo-colour heat maps of cell-response rates to each substrate exhibited greater than 5% increased fluorescence intensity before and after stimulation with 10 µM bombykal. The blue arrows in (*a*) indicate fluorescence intensity before odorant stimulation and correspond to the ‘before’ heat maps shown in (*b*). The red arrows in (*a*) indicate fluorescence intensity change peaks induced by stimulation with 10 µM bombykal and correspond to the ‘bombykal (10 µM)’ heat maps. All scale bars: 100 µm.

**Figure 3.**
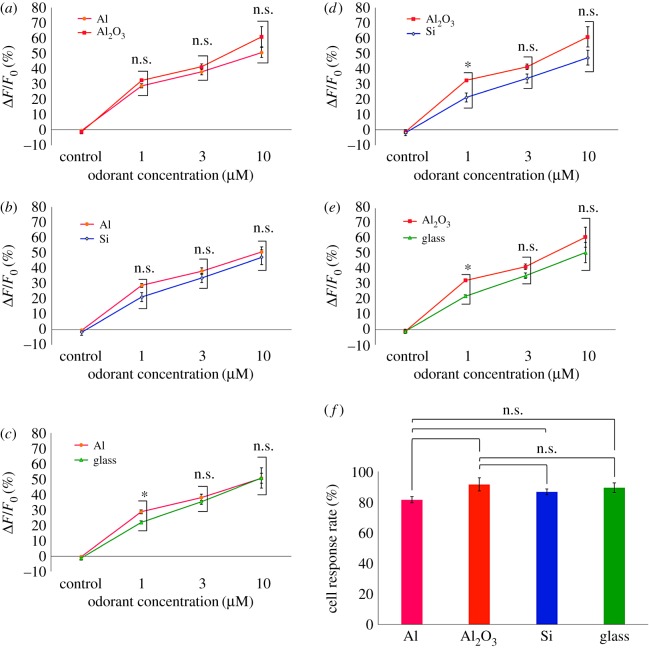
Average dose-dependent response comparisons between BmOR3 cells bound to aluminium-sputtered silicon substrates and (*a*) Al_2_O_3_-sputtered silicon substrates, (*b*) bare silicon substrates and (*c*) 12 mm diameter cover glasses. Data represent means ± SEMs of fluorescence intensity changes in all cells (*N* = 3 individual tests, Welch's *t*-test: n.s., not significant, *p < 0.05). Average dose-dependent response comparisons between BmOR3 cells bound to Al_2_O_3_-sputtered silicon substrates, (*d*) bare silicon substrates and (*e*) 12 mm diameter cover glasses. Data represent means ± SEMs of fluorescence intensity changes in all cells (*N* = 3 individual tests, Welch's *t*-test: n.s., not significant, *p < 0.05). (*f*) Comparisons of the cell-response rates on each substrate exhibited greater than 5% increased fluorescence intensity during 10 µM bombykal stimulation. Data represent means ± SEMs of fluorescence intensity changes of BmOR3 cells (*N* = 3 individual tests, Welch's *t*-test: n.s., not significant).

Cell-activity measurements using aluminium-based electrodes help us to develop low-cost biosensor systems based on the commercial CMOS foundry process by eliminating the need for biocompatible coatings. In OSFETs, Sf21 cells expressing insect ORs, such as BmOR3 cells, should function as odorant sensor elements on aluminium-based electrodes. To verify the viability of BmOR3 cells, we conducted calcium imaging of the cells on four types of substrates. The dose–response of BmOR3 cells towards bombykal doses ranging from 1 to 10 µM on each type of substrate was compared. Typical dose–response profiles of BmOR3 cells are shown in figure 2*a*. Pseudo-colour heat maps before and after 10-µM bombykal stimulation are shown in figure 2*b*. We were concerned that the toxicity of aluminium-based materials would cause abnormal fluorescence intensity changes in the BmOR3 cells; however, these experiments indicated that the dose-dependent responses of BmOR3 cells were consistent with those in previous studies, and the cells exhibited fluorescence intensity changes of more than 5% in entire images. We then compared the fluorescence intensity changes of BmOR3 cells on aluminium-based materials with those on the silicon substrates and cover glasses. The fluorescence intensity changes on aluminium-sputtered substrates were nearly the same as those on Al_2_O_3_-sputtered layers (figure 3*a*). To eliminate an effect of substrate thickness, we measured the fluorescence intensity changes of BmOR3 cells on bare silicon substrate, because the cells were seeded directly on 12 mm cover glasses in previous experiments (figure 3*b*). Fluorescence intensity changes on 12 mm cover glasses were measured as the control (figure 3*c*). We confirmed that the cells on aluminium-sputtered silicon substrate exhibited fluorescence intensities similar to or higher than those on bare silicon substrate and cover glasses. Al_2_O_3_ has previously been used in OSFET as an electrode coating, and thus, understanding the cellular state differences between the coatings will provide alternative electrode material options. The fluorescence intensities of BmOR3 cells on Al_2_O_3_-sputtered silicon substrate were similar to or higher than those on bare silicon substrate and cover glasses (figure 3*d*,*e*). Next, the cell-response rates of BmOR3 cells on aluminium-based materials were compared with those on other substrates. Over 80% of BmOR3 cells on aluminium-sputtered silicon substrates exhibited a larger than 5% increase in fluorescence intensity when stimulated with 10 µM bombykal, and the response rates were nearly the same as those on other substrates (figure 3*f*). The response rates of cells on Al_2_O_3_-sputtered silicon substrates were also nearly the same as those on bare silicon and cover glasses.

Additionally, we evaluated the cell growth of Sf21 and HEK293T cells on aluminium-sputtered substrates to evaluate any toxic effects of the aluminium. Sf21 and HEK293T cells were seeded on aluminium-sputtered substrates and on plastic cell-culture dishes as a control. Sf21 cells showed no difference in growth on aluminium substrates and plastic dishes. By contrast, the cell-adhesion areas of HEK293T cells on aluminium substrates were not uniform, and cell growth was slower than that on plastic dishes (electronic supplementary material, figures S2, S3 and S4).

### 3.2. Bird's-eye and top-side images of BmOR3 cells

**Figure 4.**
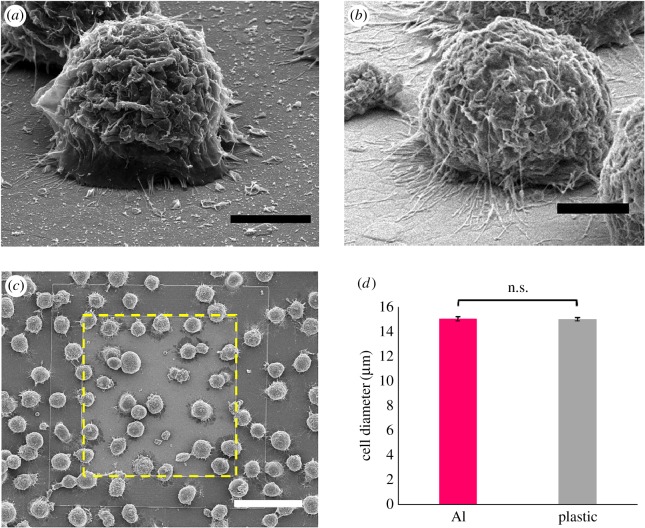
(*a*) Bird's-eye SEM images of BmOR3 cells on an Al_2_O_3_-sputtered extended-gate electrode and (*b*) on aluminium extended-gate electrode. (*c*) Top-side SEM image of the 100 × 100 µm^2^ aluminium extended-gate electrode and SiO_2_-insulator with BmOR3 cells (yellow-dashed box surrounds the sensing area). (*d*) Sf21 cell diameter comparison on the aluminium-sputtered substrate and on the plastic cell-culture dish. Data represent the means ± SEMs of the cell diameters of them (*N* = 164 for the cells on the aluminium-sputtered substrate and *N* = 190 for the cells on the plastic cell-culture dish, Welch's *t*-test: n.s., not significant). Scale bars: (*a*) 5 µm, (*b*) 5 µm and (*c*) 50 µm.

We investigated the cellular state and shape on aluminium-based extended-gate electrode surfaces to determine the viability of Sf21 cells on aluminium-based materials, which are thought to have toxic effects. BmOR3 cells on Al_2_O_3_-sputtered and aluminium extended-gate electrodes are shown in figure 4*a* and *b*, respectively. The top-side SEM images of the aluminium extended-gate electrode with BmOR3 cells are shown in figure 4*c*. The SEM images revealed that BmOR3 cells were tightly attached on both types of layers and that the cells on the aluminium extended-gate electrodes did not flee to the SiO_2_-insulator areas.

We then investigated shrinkage or expansion of living Sf21 cells on aluminium-sputtered silicon substrates and plastic dishes. Measurements made with ImageJ suggested that there were no significant differences in diameter between the 164 cells on the aluminium layers and the 190 cells on the plastic dishes (figure 4*d*).

### 3.3. Cross-sectional images of BmOR3 cells on Al_2_O_3_ layers

To observe and analyse the cleft distances between BmOR3 cells and Al_2_O_3_ layers, we developed cross-sectional specimens using the CP. Previously, a microtome method or a focused ion beam was used for specimen preparations of cell–device interfaces [9,16,24]; however, the tip of a microtome can easily snap off when cutting a device chip, and the width of a cross-sectional area of a focused ion beam is limited to several tens of square micrometres [25]. Therefore, the CP, which mainly consists of a specimen, a shielding plate, an ion gun and a broad argon ion beam, was chosen to prepare high-quality cross-sectional specimens for SEM with broad observation areas [25]. The cross sections were observed by SEM (JSM-7800F; JEOL) at a 5-kV accelerating voltage (spatial resolution: 15 kV: 0.8 nm; 1 kV: 1.2 nm).

**Figure 5.**
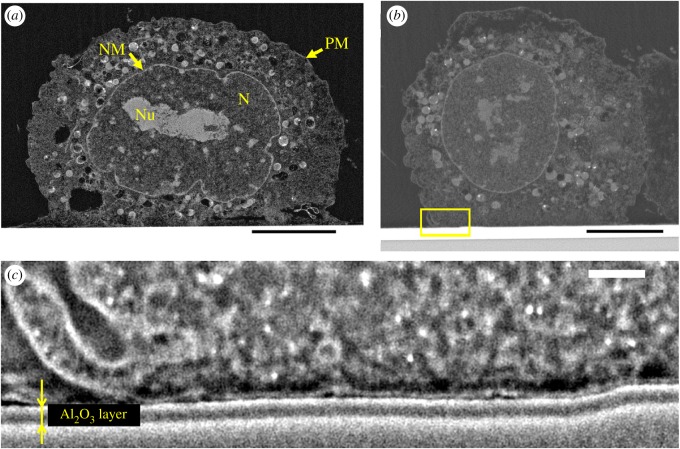
(*a*) Intracellular structures of BmOR3 cells on the Al_2_O_3_ layer. The structures were identified according to previous TEM observations of Sf9 cells [26,27]: plasma membrane (PM), nucleus (N), nucleolus (Nu) and nuclear membrane (NM). (*b*) Whole image of BmOR3 cells on the OSFET electrode with an Al_2_O_3_ layer. (*c*) Magnified view of the area in the yellow rectangle in (*b*). The thickness of the Al_2_O_3_ layer in (*c*) was approximately 63 nm (average of the thicknesses of three regions). Scale bars: (*a*) 5 µm, (*b*) 5 µm and (*c*) 200 nm.

The cross-sectional images allowed observing the intracellular structures of BmOR3 cells on Al_2_O_3_-sputtered substrates (figure 5*a*). We then observed the BmOR3 cells on OSFET electrodes. Owing to the broad processing range of the CP, we were able to develop specimens to examine the interface between two BmOR3 cells (cell A and B in the following) on one OSFET electrode with an Al_2_O_3_ layer. An overall cross-sectional image of a BmOR3 cell attached to an Al_2_O_3_ layer for cleft analysis is shown in figure 5*b*, a magnified view of the attachment interface of the same cell is shown in figure 5*c*. We found that the specimen preparation process using the CP preserved the plasma membranes of the BmOR3 cells, the planar Al_2_O_3_ layer and their interface. In this study, we have taken nine cross-sectional images of BmOR3 cells on the sputtered Al_2_O_3_ layer, out of which seven were tightly attached on Al_2_O_3_ layers. Then, we selected two cells to conduct elaborate analysis of their adhesive interface (electronic supplementary material, figure S5). Also, we did not consider the cells that were distant from Al_2_O_3_ layers due to the cutting position.

**Figure 6.**
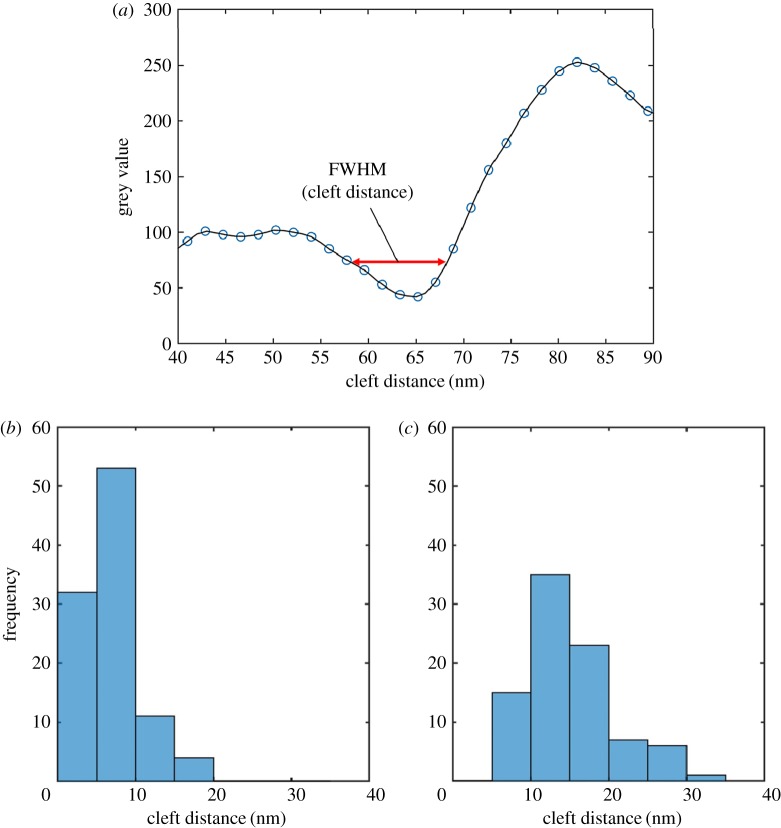
(*a*) FWHM value and cleft distance profile for the interface between the BmOR3 cells and Al_2_O_3_ layer in figure 5*c*. (*b*,*c*) Histograms showing the cleft distance distributions of BmOR3 cells on the same Al_2_O_3_ layers (analysed points in cell A, *N* = 100 and cell B, *N* = 87). (*b*) The histogram of the cleft distance distribution in figure 5*c*.

To estimate the cleft distances between BmOR3 cells and Al_2_O_3_ layers, FWHM values of sharp brightness value decreases were calculated from the plot profile. An example of a FWHM value and a plot profile are shown in figure 6*a*. Figure 6*b*,*c* show histograms of cleft distance distributions of cells A and B on the same Al_2_O_3_ layer. One hundred points of cell A and 87 points of cell B were analysed. For example, the histogram of cell A indicated that 32.0% of cleft distances were 5 nm or less, and 53.0% were 10 nm or less. The average cleft distance between cell A and the Al_2_O_3_ layer was 6.2 ± 0.4 nm (table 1). In the case of cell B, 57.4% of cleft distances were no greater than 15 nm, and there were no cleft distances greater than 35 nm. The average cleft distance of the two cells was 10.3 ± 0.5 nm. We also evaluated brightness value of free and attached parts of the membrane of the cell A to confirm that they were attached to the Al_2_O_3_ layer (electronic supplementary material, figure S6).

**Table 1.** Comparisons of the percentage of cleft distance between two BmOR3 cells and the Al_2_O_3_ layers, and their average cleft distances (analysed points, *N* = 100 of cell A and *N* = 87 of cell B).

cells	*d* ≤ 5 nm (%)	*d* ≤ 10 nm (%)	*d* ≤ 15 nm (%)	*d* > 15 nm (%)	average cleft distance (nm)
cell A	32.0	53.0	11.0	4.0	6.2 ± 0.4
cell B	—	17.2	40.2	42.5	14.9 ± 0.6
cell A and B	17.1	36.4	24.1	22.5	10.3 ± 0.5

## 4. Discussion

### 4.1. Adhesive interfaces between BmOR3 cells and aluminium-based materials

HEK293 cell–device and neural cell–device interfaces have been previously characterized by various techniques such as fluorescence interference-contrast microscopy, which has a high resolution of vertical distances [8,28,29], or transmission electron microscopy (TEM) [7,9,16], and the reported cleft widths fell within the range of 10–100 nm. This study investigated the adhesive interfaces between BmOR3 cells and Al_2_O_3_ layers with specimen preparation using the CP. The major finding of cross-sectional observations and image analysis in this study is that, beyond our initial expectations, the average cleft distances between BmOR3 cells and Al_2_O_3_ layers measured as FWHM values were shorter than those previously measured by using fluorescence interference-contrast or TEM.

**Figure 7.**
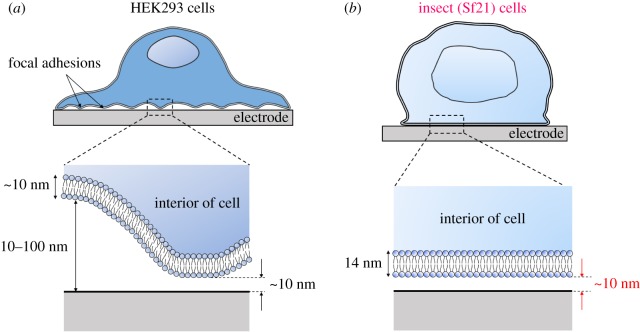
(*a*) Schematic of previously reported cell adhesion with the formation of focal adhesions on a planar electrode and a magnified view of their cell–electrode interface. A typical thickness of the plasma membrane of cells is approximately 10 nm. (*b*) Schematic of cell adhesion of insect (Sf21) cells on a planar electrode and a magnified view of their cell–electrode interface based on observations and analysis of BmOR3 cells in this study. The thickness of the plasma membrane of a BmOR3 cell on the Al_2_O_3_ layer was approximately 14 nm (average of the thicknesses of three regions based on FWHM values).

Various types of cells, including fibroblasts, epithelial cells, endothelial cells and platelets, are known to form focal adhesions when they attach to substrates [14]. In previous observations, focal adhesions between plasma membranes and substrates were typically 10–20 nm [15], and 5–20% of the close adhesion points (cleft distances no greater than 10 nm) of HEK293 cells on substrates were thought to be focal adhesion points [9]. By contrast, our analysis demonstrated that 85.0% of cleft distances of cell A fell within the range of 0–10 nm. The cleft distances of cell B were wider than those of cell A; however, the majority were still under 15 nm. These results indicated that the large areas of the bottom surfaces of BmOR3 cells were attached to electrode surfaces tightly. The average cleft distance of the two BmOR3 cells (187 analysed points) on the same Al_2_O_3_ layer was approximately 10 nm. Figure 7*a* shows a schematic of a previously reported cell adhesion with the formation of focal adhesions on a planar electrode, and figure 7*b* shows a schematic of cell adhesion of an Sf21 cell on a planar electrode based on observations from and analysis of this study. This result demonstrated that the cleft distances of Sf21 cells expressing insect ORs were obviously shorter than previous results predicted, and that the adhesion state could be different from that of other types of cells. The close adhesion points that were assumed to be focal adhesions in HEK293 cells [9] were not observed in Sf21 cells under the experimental conditions of this study. To improve cell adhesion and electrical coupling between neurons and sensing-device surfaces, spine-shaped gold microstructures protruding from flat substrates were developed [7]. By contrast, our results suggested that Sf21 cells expressing insect ORs could be tightly attached to the planar device surfaces without structural improvements. In figure 5*c*, there were black areas above the plasma membranes of BmOR3 cells. These areas might have been caused by specimen preparation; however, the plasma membrane was clearly preserved and attached to the Al_2_O_3_ layer. Therefore, we concluded that these areas had no effect on cleft distance observations. We tried to observe and measure BmOR3 cells on aluminium layers; however, we could not distinguish plasma membranes or clear brightness value decreases (electronic supplementary material, figure S7).

### 4.2. Electrical model of odour-sensitive field-effect transistor

The observed adhesion state of BmOR3 cells suggests that the electrical model of the OSFET is different from that of previously proposed FET. In this study, the average cleft distance of BmOR3 cells was approximately 10 nm. Previous electrical models of cell–device interfaces were thought to accurately describe the cleft between cells and silicon chips because of their certain degree of cleft distance [30–32]. The cleft filled with ionic solution generates resistance, termed ‘seal resistance’. The voltage formed over the resistance modulates FET gate voltage [32].

By contrast, our SEM study suggests that the electrical model of the OSFET might be driven by capacitive coupling. The OSFET uses Sf21 cells expressing insect ORs along with OR co-receptors, which are ligand-gated ion channels, causing inward non-selective cation influx (including Na^+^ and Ca^2+^) when bound to specific odorant molecules [33]. This leads to increased intracellular electrical charges when the insect ORs respond to specific odorants, and it may directly modulate the drain current of FETs. We previously detected increases in the drain current of the OSFET when Sf21 cells expressing insect ORs responded to specific odorants [3]. According to this model, tightly coupled plasma membranes of Sf21 cells expressing insect ORs and device surfaces could directly transfer the effect of electrical charge increases inside cells to FETs. Then, the FET channels increase, leading to an increase in drain current. The observations in this study support previous measurement results of the OSFET.

### 4.3. Cellular state on aluminium-based materials

Knowledge of the cellular state of Sf21 cells on aluminium-based materials could benefit the development of simple cost-effective bio-hybrid electronic odorant sensor systems. We generally sputtered Al_2_O_3_ layers onto extended-gate electrodes of the OSFET for stable surface potentials. Calcium imaging of BmOR3 cells indicated that the cells on aluminium-sputtered silicon substrate exhibited fluorescence intensity changes equivalent to those on Al_2_O_3_-sputtered silicon substrate, bare silicon substrate and 12 mm diameter cover glasses without affecting their sensitivity, cell-response rate or dose–response profile. In 1 µM bombykal stimulation, the fluorescence intensity changes of BmOR3 cells on aluminium-sputtered substrate were larger than those on 12 mm diameter cover glasses (Al, 28.8 ± 1.3%; glass, 22.0 ± 1.0%; *p* = 0.015). In some cases, the fluorescence intensity changes of BmOR3 cells on Al_2_O_3_-sputtered substrate were higher than those on bare silicon substrate (Al_2_O_3_, 32.4 ± 1.7%; Si, 21.3 ± 2.9%; *p* = 0.046) and 12 mm diameter cover glasses (Al_2_O_3_, 32.4 ± 1.7%; glass, 22.0 ± 1.0%; *p* = 0.013). The high reflectance of aluminium or Al_2_O_3_ surfaces may increase, but in any case did not decrease, the intensity changes. The cell shapes on aluminium-based extended-gate electrodes did not change. Cell diameter comparisons indicated that there was no irregular expansion, shrinkage or collapse (an Sf9 cell is typically approximately 15 µm in diameter [34]). Cell growth and cell area comparisons between Sf21 and HEK293T cells on aluminium substrate also supported the conclusion that Sf21 cells are viable on aluminium-based materials. By contrast, HEK293T cell growth on aluminium was distinctly different from that on plastic dishes, suggesting that these cells are not suitable for use on aluminium electrodes. Sf21 and HEK293T cells are generally cultured at different pH (Sf21 cells, pH 6.4; HEK293T cells, pH 7.8); however, this study did not take into account the influence of pH of the cell-culture medium.

The experimental results indicated that Sf21 cells expressing insect ORs exhibited aluminium tolerance, despite the concern regarding toxicity [11–13]. This unique characteristic of Sf21 cells suggests that the commercial CMOS foundry process can be directly applied to Sf21 cells without inducing damage, thereby demonstrating its potential to reduce post-processing costs, including expenses for biocompatible coatings.

## 5. Conclusion

In this study, we investigated the interface between Sf21 cells expressing insect ORs and aluminium-based layers. Specimen preparation using the CP for SEM observations allowed us to analyse the detailed nanostructures of the Sf21 cell–device interfaces, and image analysis of cleft distances indicated that the majority of the bottom surfaces of Sf21 cells expressing insect ORs attached to device surfaces at distances shorter than those previously reported. These results suggested that the cells attached to the device surface by a different mechanism than focal adhesion. Additionally, this study provided the information to support that the electrical model of the OSFET could be the capacitive coupling of the plasma membranes of Sf21 cells expressing insect ORs and device surfaces, and is unrelated to ionic current in the cleft of cell–device interfaces. The results of calcium imaging and cell-growth observations led us to expect that Sf21 cells expressing insect ORs could function as odorant sensor elements on aluminium-based layers without being damaged, and that OSFETs can utilize aluminium-based electrode materials for reliable and cost-effective odorant sensor systems based on the commercial CMOS foundry process.

## Supplementary Material

Supplementary Materials

## Data accessibility

All data used in this research are included in the figures, the table and the electronic supplementary material.
