# Supplementary material for: Increasing cell–device adherence using cultured insect cells for receptor-based biosensors
Source: R Soc Open Sci. 2018 Mar 21;5(3):172366. doi: 10.1098/rsos.172366 (PMC5882746; doi:10.1098/rsos.172366)
Supplement: Supplementary Materials [file rsos172366supp1.pdf]

Supplementary Materials

Increasing cell–device adherence using cultured insect cells for receptor-based biosensors

Daigo Terutsuki,[1] Hidefumi Mitsuno,*[2] Takeshi Sakurai,[2] Yuki Okamoto,[3] Agnès Tixier-Mita,[2] Hiroshi Toshiyoshi,[2] Yoshio Mita,[3] and Ryohei Kanzaki*[2]

[1]Department of Advanced Interdisciplinary Studies, Graduate School of Engineering, and [2]Research Center for Advanced Science and Technology (RCAST), The University of Tokyo, 4-6-1 Komaba, Meguro-ku, Tokyo, Japan

[3]Department of Electrical Engineering and Information Systems, Graduate School of Engineering, The University of Tokyo, 7-3-1 Hongo, Bunkyo-ku, Tokyo, Japan

* Corresponding authors. *E-mail addresses:* mitsuno@brain.imi.i.u-tokyo.ac.jp, kanzaki@rcast.u-tokyo.ac.jp

Analysed adhesive between a BmOR3 cells and Al_2O_3 layer

We drew vertical plot profile lines at 10 pixel intervals using ImageJ macro to measure the cleft distance. Details of the measured points are shown in Figure S1 as yellow lines.

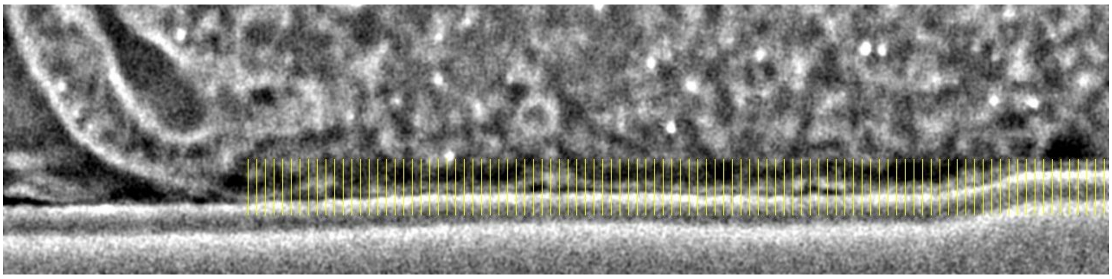

Figure S1 Plot profile lines for measuring the cleft distance between a BmOR3 cells and Al_2O_3 layer. Vertical yellow profile lines were drawn at 10 pixel intervals.

Evaluation of cell growth on aluminium

The cytotoxicity of aluminium was evaluated using trypan blue exclusion assay and Sf21 and HEK293T cells cultured on aluminium-sputtered silicon substrates. Before seeding the cells, the aluminium substrates were immersed in 70% ethanol for 1 h and then in 99.5% ethanol for 30 min for sterilization and drying. 50-µL suspensions of Sf21 cells were seeded on the aluminium substrates in 35-mm plastic cell culture dishes containing 2 mL of insect culture medium, and the cultures were incubated at 27°C. Time-dependent changes in Sf21 and HEK293T cells were measured at 0 (2 h after seeding), 1, 2, and 3 days under a bright-field microscope. At each time point, 1 mL of culture medium was removed from the cell-culture dish and 200 µL, 0.4 w/v % trypan blue solution (Wako Pure Chemical Industries Ltd., Osaka, Japan) was added to each dish.

We measured cell-adhesion areas of Sf21 and HEK293T cells cultured on aluminium-sputtered substrates to evaluate the cytotoxicity of aluminium. Sf21 and HEK293T cells were seeded separately on aluminium-sputtered substrates (test) and plastic cell culture dishes (control). Figures S2 and S3 show the time-dependent changes in the Sf21 and HEK293T cells from 0 to 3 days. Each day, samples were observed by bright-field microscopy after addition of trypan blue. Figure S4 shows the comparison of time-dependent changes in Sf21 and HEK293T cell areas on the aluminium substrates and plastic cell culture dishes. The bright-field microscopy images and the graphs indicate that Sf21 cells cultured on aluminium substrates grew and were not stained by trypan blue, similar to the cells cultured in plastic dishes. In contrast, HEK293T cell-adhesion areas on aluminium substrates were not constant, and cell growth was slower than that on plastic dishes. Moreover, some HEK293T cells cultured on the aluminium substrates were stained each day by trypan blue.

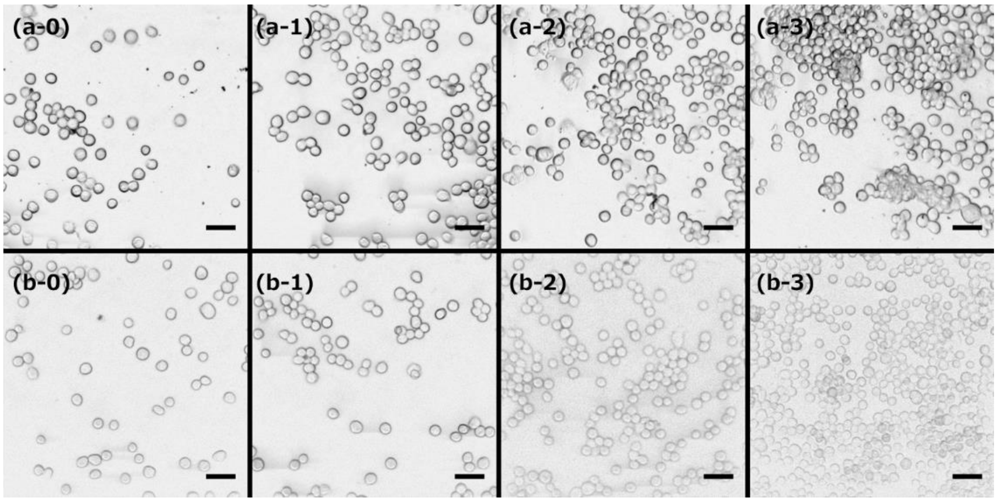

Figure S2 Time-dependent changes in Sf21 cells from 0 to 3 days. (a-0)–(a-3) the Sf21 cells on aluminum-sputtered silicon substrates after addition of trypan blue; (b-0)–(b-3) on plastic cell culture dishes after addition of trypan blue. All scale bars are 50 μm.

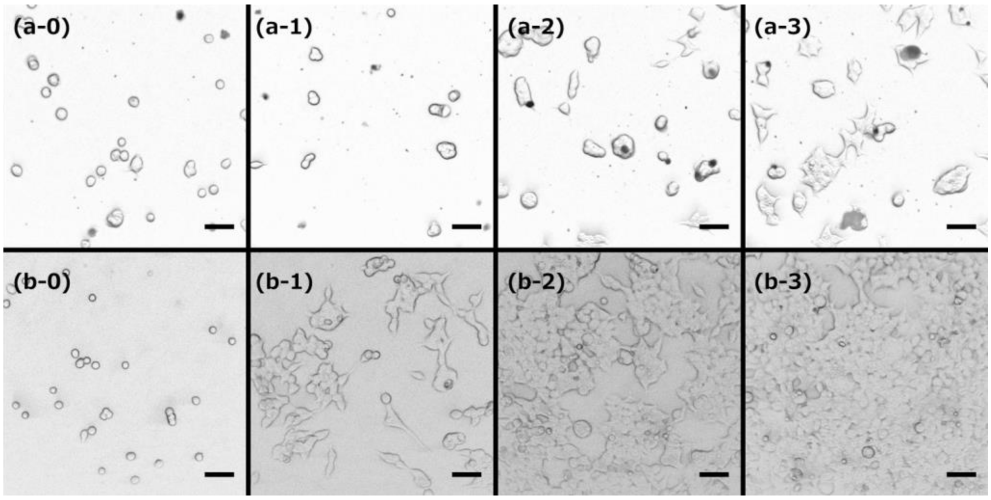

Figure S3 Time-dependent changes in HEK293T cells from 0 to 3 days. (a-0)–(a-3) HEK293T cells on aluminum-sputtered silicon substrates after addition of trypan blue; (b-0)–(b-3) on plastic cell culture dishes after addition of trypan blue. All scale bars are 50 μm.

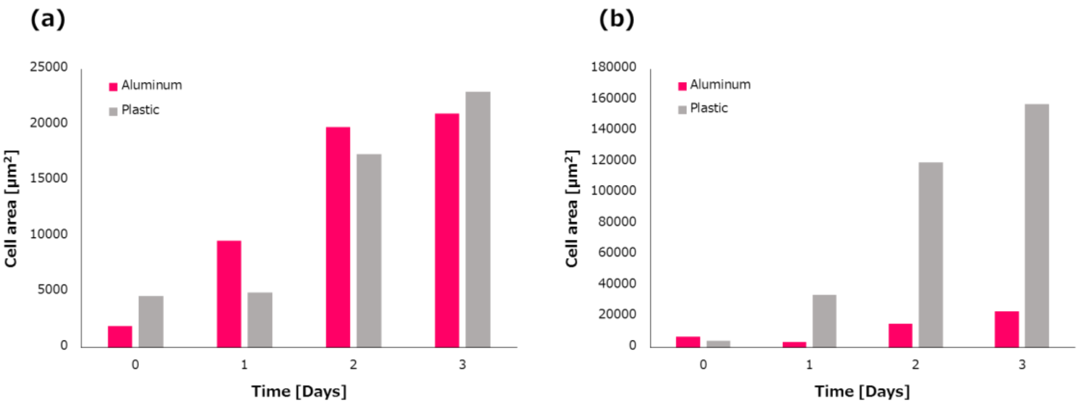

Figure S4 Comparison of time-dependent changes in Sf21 cell areas and HEK293T cell areas on the aluminum substrates and the plastic cell culture dishes. (a) Sf21 cells: the upper right-hand quarter of the bright field microscopy images (204.8 × 204.8 μm^2) in Figure S2 were measured; (b) HEK293T cells.

All cross-sectional SEM images of attached BmOR3 cells on Al_2O_3 layers

In this study, we have taken nine cross-sectional SEM images of BmOR3 cells cultured on sputtered Al_2O_3 layers. One cross-section included multiple cells (Figure S5 (a)), suggesting that a majority of the cells were tightly attached. BmOR3 cells on sputtered Al_2O_3 layers on Si substrates (Figure S5 (b)–(h)) or extended-gate electrodes of the OSFET (Figure S5 (i) and (j)) are shown. These SEM images indicated that a majority of the cells were tightly attached onto Al_2O_3 layers except the cells shown in Figure S5 (c) and (e). We conducted elaborate analysis of adhesive interface of two cells (cell A and B).

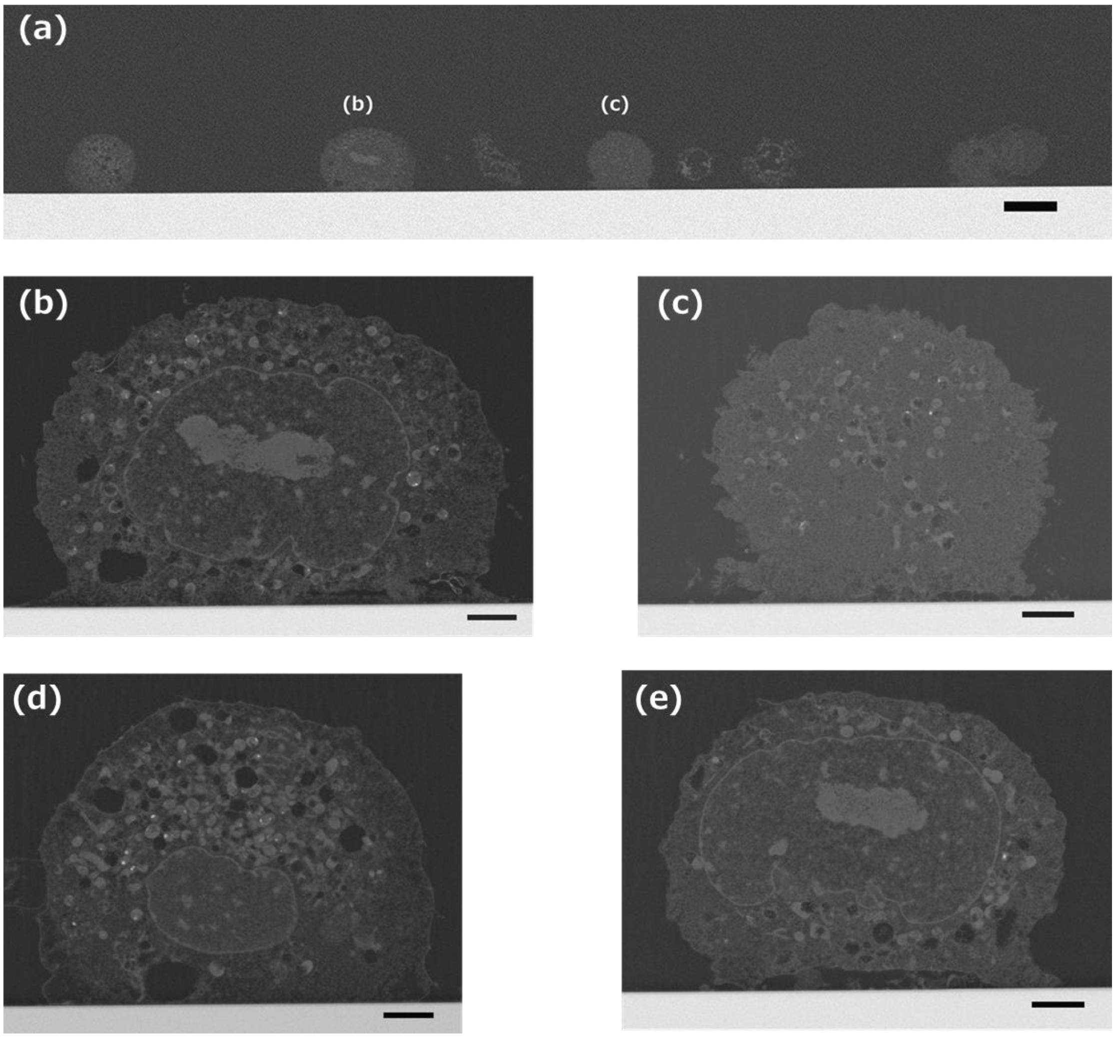

Figure S5 Cross-sectional SEM images of BmOR3 cells attached on the sputtered Al_2O_3 layers. (a) indicated that one cross-section included multiple cells ((b) and (c)). Scale bars, (a) 10 μm, (b)–(e) 2 μm.

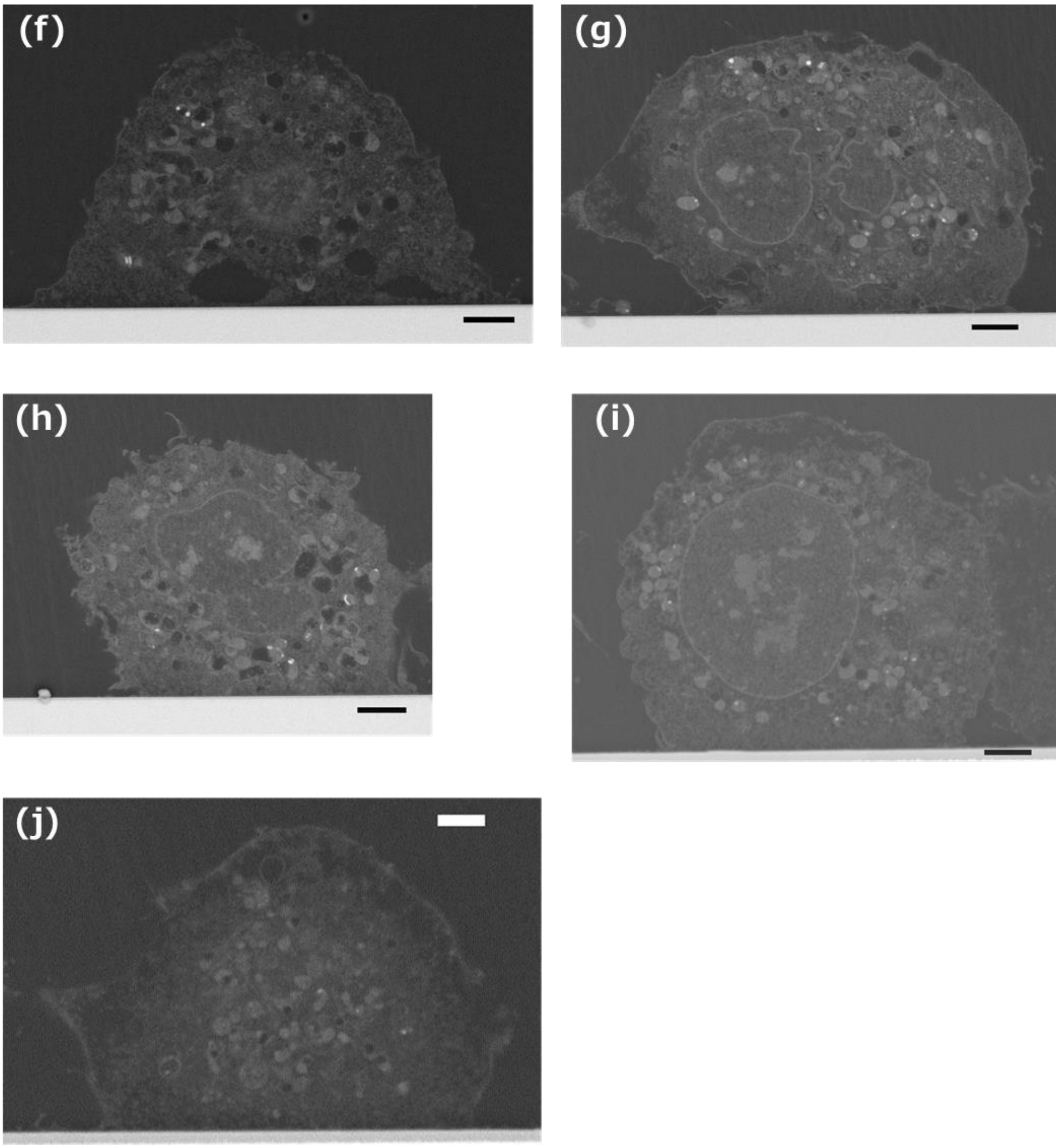

Figure S5 (continued) Cross-sectional SEM images of BmOR3 cells attached on the sputtered Al_2O_3 layers. All scale bars are 2 μm.

Brightness value analysis of cell membrane using ImageJ

To confirm that the membrane of the BmOR3 cell was attached to the Al_2O_3 layer, we measured brightness value of the free membrane and attached membrane of the cell A using ImageJ software (Figure S6). Results showed that floating parts of the cell had long cleft distances (Line1 to 3 of Figure S6) and attached parts of the cell had short cleft distances (Line4 to 5 of Figure S6). Therefore, we concluded that the cell membrane was certainly attached on the device surface.

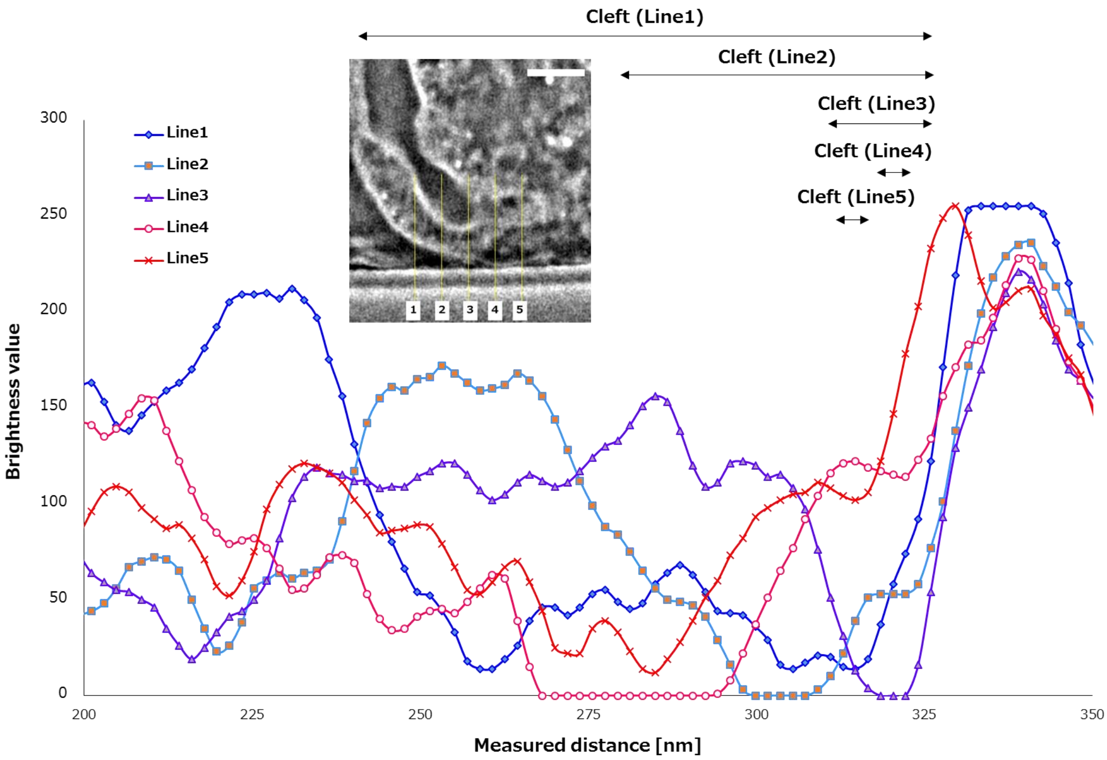

Figure S6 Brightness value analysis of the attached BmOR3 cell membrane. The analyzed BmOR3 cell is shown. Five yellow lines indicate measured distance. Vertical yellow profile lines were drawn at 50 pixel intervals. The width of the profile lines was set to 1 pixel (= 1.86 nm). Double-headed arrows are indicated to understand approximate cleft positions. Scale bar is 200 nm.

Cross-sectional SEM images of BmOR3 cells on aluminium layer

To observe and analyse cleft distances between BmOR3 cells and aluminium layers, we developed cross-sectional specimens using a cross-section polisher similar to that for the cells on Al_2O_3 layers (Figure S7 (a)). However, we could not distinguish the plasma membranes of BmOR3 cells (Figure S7 (b)) and brightness value decreased between the cell–device interfaces.

(a)

(b)

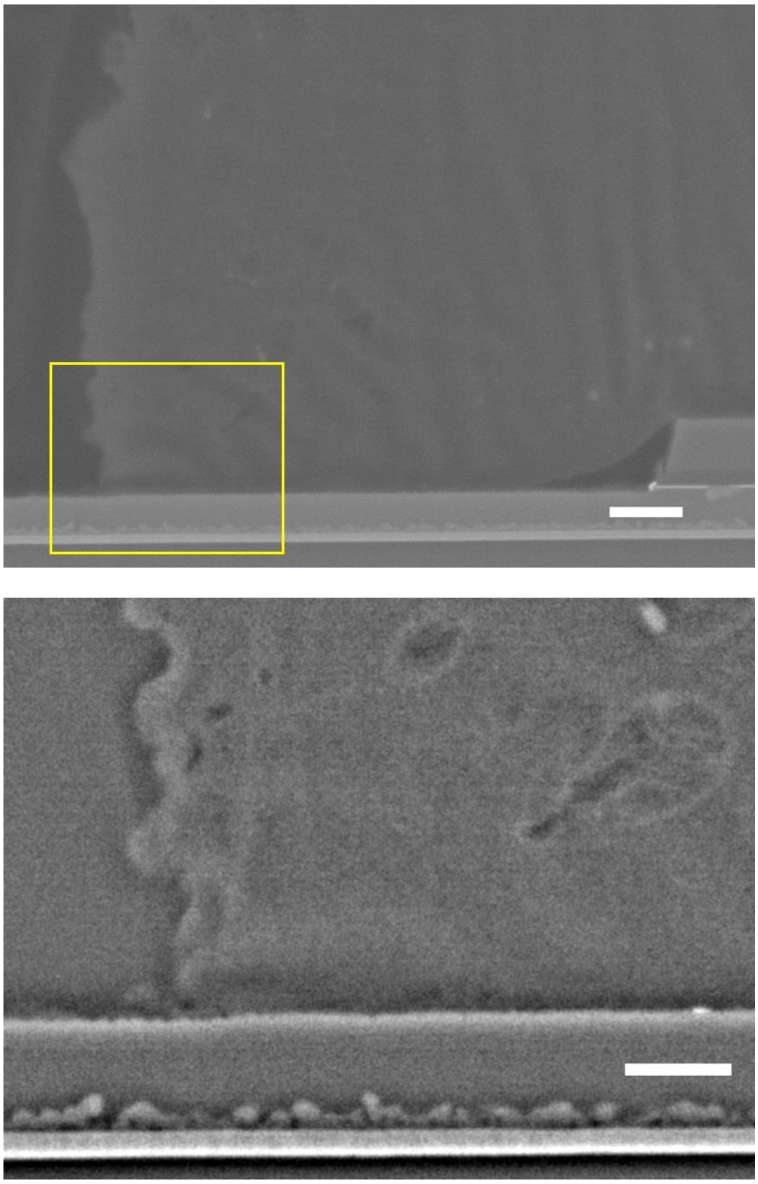

Figure S7 (a) Whole image of the BmOR3 cells on the aluminum layer. (b) Magnified view of the area inside the yellow rectangle in (a). Scale bars, (a) 1 μm, (b) 500 nm.
